# Sea-Sickness

**Published:** 1881-09

**Authors:** 


In crossing the sea with a large party of his own patients, a physician naturally interests himself in the symptoms of, and the effect of treatment upon, that invariable element of an ocean trip, sea-sickness. Dr. Beard, of New York, has lately written a pamphlet upon this complaint, and his chief weapon against its inroads is the potassium bromide, with which he advises physicians to intoxicate their patients before they go on board the steamer. This bromidism is then to be kept up during the passage, and thus, thinks Dr. Beard, sea-sickness may be kept at bay. I regret that I cannot endorse this opinton and this advice. In the first place, I can hardly believe that any physician of calm judgment would place delicate women under such overwhelming influence of bromide for several days before they go to sea and continue it during the voyage. Secondly, I do not believe such treatment would overcome or prevent sea-sickness. Given in proper doses, while at sea, the bromides in my hands effected nothing whatever. Nitrate of amyl is another remedy suggested by others as well as by Dr. Beard. I used it in strong doses. It not only did no good whatever, but its penetrating, to many, nauseating, odor disturbed the stomachs of those who otherwise, perhaps, would not have been so ill. Dr. Beard thinks the galvanic current an excellent remedy in sea-sickness, but says its use at sea is impracticable. By cutting small openings in the cover of a Hall's French battery for the passage of the wires and afterward suspending the box, I found no difficulty whatever in passing the Faradic current through the stomachs of my patients, the positive pole being placed in the epigastric region, the negative over the base of the brain. In one case this procedure seemed to effect a temporary relief. In all others it failed to accomplish any good whatsoever. The citrate of coffeine is also recommended. Its effects are *nil.* In short, I believe there are only two remedies for sea-sickness, and they are rather palliative than curative, but they do lessen the duration of the attack. One of these is *fresh air* taken in a supine position on deck, the other is indomitable *pluck.* If patients cannot be kept perfectly warm on deck it is better that they keep their berths. Cold at sea is extremely exhaustive. In one of my patients it caused an attack of neuralgia which exposed the lady to serious danger. Food of simple character, such as biscuit, oatmeal porridge, baked potatoes, well salted, and a little black coffee without sugar, will gradually accustom the stomach to food of more solid character. But the rule should be that if such simple nourishment be ejected by the stomach, a fresh quantity should at once be taken. It is not necessary to say that lemons, oranges (and perhaps apples,) are indispensible.

A small mustard plaster over the epigastric region is sometimes a comfort, and hot water at the feet is a delight. But the *medical* treatment of sea-sickness is a snare and a delusion. It not only does no good, but I believe rather increases than assuages that feeling of unspeakable disgust, which led Charles Dickens to say on the first day out, that he was afraid he should die, and on the second, that he was afraid he should not.

In several voyages across the Atlantic I have as yet failed to meet a ship's surgeon who had any faith in medical remedies against sea-sickness. These gentlemen are as heretical in this direction as the veriest old sea-dog before the mast. They try to sympathize with sea-sick passengers but are quite ready to confess their absolute ignorance of effectual remedies. I am inclined to believe, however, that the doctors on ocean steamers are not overwhelmed by ambition. For the most part they are genial, pleasant fellows, ready and willing to do one a kindness. They go back and forth between two ports, year after year, quite satisfied, apparently, with their lot and their salary of four or five hundred dollars. They are generally unmarried. Living costs them nothing. They have a good table, and their principal outlay is for the clothes they wear.—*Foreign Cor. Chicago Med. Jour. and Examiner.*

